# Challenges and Opportunities in Multi-Omics Data Acquisition and Analysis: Toward Integrative Solutions

**DOI:** 10.3390/biom16020271

**Published:** 2026-02-09

**Authors:** Christopher L. Hemme, Janet Atoyan, Ang Cai, Chang Liu

**Affiliations:** 1 Department of Biomedical and Pharmaceutical Sciences, College of Pharmacy, University of Rhode Island, Kingston, RI 02881, USA; hemmecl@uri.edu; 2 RI-INBRE Molecular Informatics Core, College of Pharmacy, University of Rhode Island, Kingston, RI 02881, USA; jatoyan@uri.edu; 3 Rhode Island IDeA Network of Biomedical Research Excellence (RI-INBRE), Kingston, RI 02881, USA; 4 Proteomics Facility, College of Pharmacy, University of Rhode Island, Kingston, RI 02881, USA

**Keywords:** multi-omics, systems biology, single-cell omics, spatial omics, data integration, biomarker discovery, computational analysis

## Abstract

In this perspective, we discuss the current challenges and opportunities in multi-omics, a rapidly evolving approach that integrates multiple molecular layers to advance our understanding of complex biological systems. As biomedical research moves toward precision medicine, the ability to correlate genotype, phenotype, and environmental contexts has never been more critical. Multi-omics enhances biomarker discovery and elucidates regulatory networks underlying health and disease. The dominant scientific paradigm for over a century was to take a reductionist approach, studying individual molecular components in isolation or as simplified systems. The advent of omics technologies in the 1990s enabled a systems paradigm, allowing holistic analyses of molecular networks. These early systems studies were constrained by technology and methodology to bulk tissue measurements and single-omics analyses. Recent advances in single-cell and spatial omics, high-throughput proteomics and metabolomics, cloud computing, and artificial intelligence now allow high-resolution, spatially contextualized multi-omics analyses. Despite these gains, challenges in data analysis and interpretation remain, including high dimensionality, missing or incomplete data, multiple batch effects, and method-specific variability. Emerging strategies—such as paired data collection, staged or joint integration, and latent factor or quasi-mediation frameworks—offer promising solutions, positioning multi-omics as a transformative tool for elucidating complex mechanisms and guiding personalized medicine. Continued refinement of these approaches may further enhance the utility of multi-omics for understanding complex biological systems.

## 1. Introduction

Multi-omics refers to the integrated analysis of multiple molecular layers within a biological system, providing a more comprehensive view of complex processes than single-omics studies alone [1]. By combining data across molecular layers and incorporating environmental or clinical data, multi-omics increases statistical power for biomarker discovery and for elucidating mechanisms linking genotype to phenotype [2]. Multi-omics datasets are high-dimensional, often comprising thousands of features, which present substantial analytical challenges [2,3,4]. The integration of multi-omics with non-omics data—such as clinical records or environmental exposures—further enhances biological insight through a multimodal approach but also adds complexity to data analysis [5]. However, such multimodal models of phenotypes are essential from pharmacy practice and precision medicine perspectives. In this perspective, we summarize the status and types of multi-omics studies, discuss key challenges in multi-omics analysis, with an emphasis on effective data integration and multimodal mediation strategies, offering insights from both technical and bioinformatics perspectives.

## 2. Defining Multi-Omics and Its Core Study Types

Multi-omics represents an integrative analytical approach that concurrently examines multiple molecular layers, such as the genome, transcriptome, proteome, and metabolome, to construct a holistic model of biological systems [1,2,6,7,8]. By synthesizing these disparate data types, researchers can uncover the complex, mechanistic pathways that link genotype to phenotype, significantly enhancing the power to identify robust biomarkers [5,9,10,11]. However, the promise of multi-omics is tempered by substantial computational challenges, as the analysis of these high-dimensional, heterogeneous datasets requires sophisticated bioinformatic workflows [12,13,14]. The development of robust, standardized pipelines is therefore critical, offering researchers a powerful foundation to accelerate discovery across diverse biological domains.

**Figure 1:**
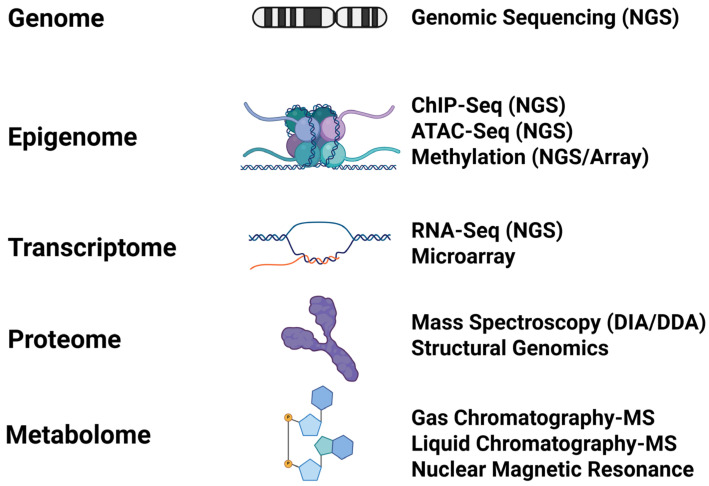
Common Molecular Layers, the Molecules Studied, and Associated Omics Technologies Used to Generate and/or Analyze the Layer. Each omics layer may be described at the bulk tissue or single-cell level. Created in BioRender. Hemme, C. (2025) https://BioRender.com/7dvj5u1.

For most of the history of science, limitations in instruments and analytical techniques required researchers to adopt a reductionist paradigm for biomedical research. In such a paradigm, complex systems were reduced to their component parts and studied in isolation or in simplified, well-defined systems. While this strategy was highly successful in elucidating molecular mechanisms of disease, it was not well-suited to identifying synergistic effects that naturally emerge in a unified system. With the advent of omics in the 1990s, researchers were able to adopt a systems paradigm that allowed for analysis of molecular systems as a whole [4,15,16,17,18,19] (Figure 1). In conjunction with the reductionist paradigm, this new era of “omics” allowed significant advances in understanding biological systems, particularly regarding human health, particularly when coupled with genome-wide association studies linking genetic variants to disease [1,20,21,22,23]. However, like the reductionist paradigm, the systems paradigm was limited by technology to bulk analysis of tissues, and typically, omics levels were analyzed individually, even though it was recognized that cells within tissues displayed significant heterogeneity [24,25,26,27]. Recent improvements in single-cell and spatial biology, as well as advances in cloud computing and artificial intelligence, have now made it possible to conduct multi-omics analyses on individual cells of complex organisms and to place that information in a spatial context, resulting in spatially oriented whole system analyses [28,29,30,31].

**Figure 2:**
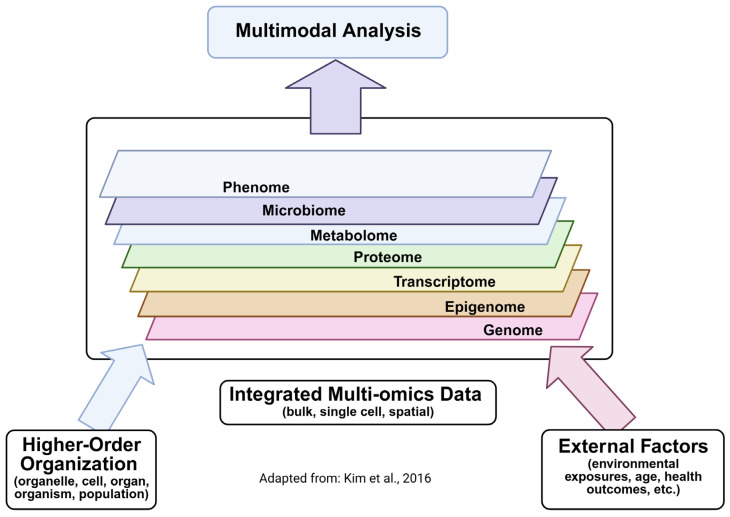
Multi-omics Hierarchy Showing Integration of Multiple Omics Layers and Incorporation of Non-Omics Data. Created in BioRender. Hemme, C. (2025) https://BioRender.com/fd4zb47. Adapted from Kim et al. 2016 [6].

Early in the omics era, researchers recognized the value of correlating multiple omics levels into unified systems (Figure 2). Systems biology is an attempt to correlate multiple omics levels together for the purpose of building mathematical models of organisms and metabolic pathways that could be used to test predictions or to engineer metabolic networks [32]. While systems biology had early successes, it was limited by several technological realities. For instance, biological and industry factors led to a rapid increase in nucleotide sequencing technology and associated omics methods such as RNA-seq, while proteomics and metabolomics methods lagged [33,34]. Early efforts at systems biology tended to focus on mathematical modeling of microbes for metabolic engineering and synthetic biology, as microbes were easier to model than complex organisms. In recent years, advancements in mass spectrometry (MS) technologies have allowed the fields of proteomics and metabolomics to reach a comparable level of analytical maturity [35], and advanced computing resources have become more accessible for average researchers, which has prompted a renewed emphasis on studying complex systems [36].

Like systems biology, multi-omics seeks to correlate multiple omics levels together to gain a better understanding of complex systems [6,7,8,36,37]. The goal of a multi-omics experiment is to increase the statistical power of data by drawing power from across multiple omics layers for the purposes of identifying biomarkers and elucidating the mechanisms linking genotype to phenotype. Multi-omics can be combined with non-omics datasets such as clinical information, health informatics, and environmental exposure data to create multimodal studies that seek to understand biological systems in the context of their environments [38,39]. A multi-omics experiment can encompass any combination and type of omics levels, but typically involves two or more of the following:

- Genome—The entire complement of genetic material in the cell, including chromosomes, plasmids, or other non-chromosomal elements. The genome is typically annotated to include protein-coding sequences (genes), non-coding regions (introns, promoters, regulatory elements, non-coding RNAs, etc.), and uncharacterized regions. The genome is generally considered to be static and represents the blueprint of the cell. Reference genomes for most model organisms have been sequenced and are available for public use [40,41,42,43,44].
- Transcriptome—The complement of transcribed genes under a given set of conditions at a given time. The transcriptome is dynamic and may be described in spatial or temporal terms depending on the experiment. Transcriptomics is the most well-defined of the functional (i.e., dynamic) omics methods [45,46].
- Epigenome—Epigenomics includes any modifications to the DNA (e.g., methylation) or histones that affect chromatin accessibility. The most common epigenomic methods are methylation, ChIP-Seq (for assessing chromatin accessibility linked to histone modification or for identifying protein-binding sites in DNA), and ATAC-seq (for assessing chromatin accessibility between nucleosomes) [47]. The epigenome has been implicated in many disease-related phenomena such as oncogenesis, aging, and toxicology [48,49].
- Proteome—The entire complement of active proteins in the cell. Proteomics is the preferred omics level for pharmaceutical science applications because proteins are typically the targets for drugs [50]. Proteomics also encompasses post-translational modifications of proteins (e.g., phosphorylation, glycosylation, etc.) and can be used to identify protein–protein networks [51,52]. Proteomics technology has improved significantly in recent years, allowing for more complete assessment of proteomes even in low-biomass samples such as biological fluids [53,54].
- Metabolomics—The entire complement of small molecules in the cell. Subsets of metabolomics include glycomics (sugars) and lipidomics (lipids) [55,56,57]. Metabolomics using radioisotope-labelled substrates can be used to assess the flow of metabolites through metabolic pathways (fluxomics) for purposes of metabolic engineering [58].
- Microbiome Omics—Omics studies of host-associated or free-living microbial communities. These methods correspond to the methods described above (metagenomics, metatranscriptomics, metaproteomics, etc.) [59]. Microbiome studies involve complex communities of multiple organisms and are increasingly included in multi-omics studies to identify molecular interactions between the host and its microbiome [42,60,61].

Although the multi-omics framework is unlimited in the possible number of integrated datasets, practical considerations mean current research is largely characterized by the simultaneous analysis of two to three molecular layers. This strategy makes the analysis more computationally manageable while still providing powerful insights into specific biological mechanisms. Representative core study types include the following:

- ChIP-Seq + ATAC Seq + Methylation—ChIP-Seq is used to analyze the effects of histone modifications on chromatin accessibility. ATAC-seq complements this analysis by analyzing free chromatin between histones. Methylation, in contrast, analyzes methylation of cytosines in DNA molecules, which can affect transcription or chromatin accessibility. In combination, these methods provide a high-level assessment of chromatin accessibility [62,63].
- Transcriptomics + Epigenomics—The epigenomics methods described above can also be combined with RNA-seq to gain a deeper understanding of gene transcription by linking chromatin accessibility to transcription. RNA-seq can also be linked to ChIP-seq for identification of DNA-binding proteins, which is a crucial method for identifying and characterizing regulatory networks [64,65]. Because of the utility of these approaches, companies have recently released several protocols for paired transcriptomic–epigenomic sample prep and analysis, which reduces technical and biological variability (see Challenges in Multi-Omics Analysis).
- Transcriptomics + Proteomics—A crucial step in the flow of biological information is translation of mRNA transcripts to active proteins. While the translation process is well-known, less clear are the multitude of post-translational modifications possible before an active protein is produced. Recent multi-omics work has clearly demonstrated a disconnect between the transcriptome and proteome due to post-transcriptional and post-translational regulatory mechanisms [1,3,66,67,68,69]. A multi-omics approach to transcription and proteomics can help elucidate these processes [70].
- Proteomics + Metabolomics—Proteins carry out many chemical reactions in the cell, which can be assessed by metabolomics. Metabolomics gives a sense of which proteins are active and how active they are [71,72]. This can be assessed directly through fluxomics, which uses radioisotope-labelled substrates to measure the flow of metabolites through metabolic pathways, which is essential for metabolic engineering [73,74,75].
- GWAS + Transcriptomics or Proteomics—Genome-wide association studies (GWAS) seek to associate genetic variants such as single-nucleotide polymorphisms (SNPs) and insertions-deletions (indels) with diseases or other phenotypes, often in the context of an external environmental exposure. The association alone, however, does not provide much information on the molecular mechanism linking the variant to the phenotype. Some variants may occur in intergenic regions, which may affect gene transcription. Other variants may occur in protein-coding genes, which may change the structure and/or activity of the translated protein. By coupling transcriptomics and proteomics to GWAS, the mechanisms by which genetic variants lead to disease phenotypes can be elucidated [76,77,78].

- Host Omics + Microbiome Omics—Interactions between the host and its microbiomes are increasingly recognized as critical to understanding and treating human health conditions such as inflammatory bowel disease or Type 2 diabetes [79]. Interactions of the microbiome with the host immune system or through metabolite production can manifest in a variety of ways, including emergence of or protection from pathogens, efficiency of digestion, and microbial metabolism. Terms such as the gut–brain axis describe how host–microbiome interactions in one part of the body can manifest in other parts of the body, either through immune system interactions or through the production of microbial metabolites such as small-chain fatty acids [79,80,81]. Increasingly, microbiome data is being included in pharmacogenomic analyses along with immunology data from the host to provide a more complete understanding of how both the host and the microbiome react to drugs [82,83]. The broad coupling of pharmacology and systems biology is often referred to as systems pharmacology [84,85]. Furthermore, multi-omics approaches are increasingly applied to infectious diseases to map host–pathogen interactions, simultaneously capturing pathogen virulence factor expression (transcriptomics) and the host immune proteome [79].

While reductionist studies of individual omics layers have yielded invaluable insights, this view of biological systems is inherently incomplete [1]. Multi-omics provides a powerful complement to reductionist research by synthesizing multiple layers to construct a more holistic model of life [20]. By strategically integrating data across these layers, we can resolve the complex causal pathways from genetic blueprint to phenotypic expression, thereby accelerating the discovery of biomarkers and therapeutic targets. The primary challenge is no longer justifying the need for integration but rather solving practical difficulties of computational and statistical synthesis of these high-dimensional networks. However, the direction of progress is clear: as these analytical hurdles are overcome, multi-omics is poised to become the central methodology for a systems-level understanding of biology. By further incorporating environmental and clinical datasets, it will provide mechanistic insights important to advance personalized medicine and better understand a comprehensive picture of molecular physiology.

## 3. Challenges in Multi-Omics Analysis

Analysis of individual omics levels presents numerous challenges. Omics datasets are high-dimensional, usually consisting of thousands of correlated features (e.g., genes, proteins, etc.) representing complex regulatory and interaction networks. These datasets are also zero-inflated, meaning a feature is missing because of technical drop-out or because the feature may not be expressed under the experimental conditions being studied [86]. Incomplete coverage and missing data are another major issue. For instance, though bottom-up proteomics has advanced considerably recently due to significant advances in Liquid Chromatography–Mass Spectrometry (LC–MS) and Gas Chromatography–Mass Spectrometry (GC–MS), data-independent acquisition still results in significant missing data within proteomics datasets [87]. Another example, genomics has been extensively characterized for protein-coding regions of DNA, yet these regions represent only a small portion of the entire genome [67]. Such incomplete coverage necessitates data processing strategies to address missing or low-quality entries. Batch effects, both technical and biological, also pose significant challenges [88]. These effects often represent technical variations unrelated to the biological factors of interest, commonly arising in both single-omics and multi-omics analyses [88]. Differences in experimental design, laboratory conditions, reagent lots, operators, and other non-biological factors can introduce unwanted variability between batches, potentially leading to misleading conclusions [89,90]. Normalization and batch effect correction are required to enable accurate cross-feature and cross-sample comparisons [91]. For multi-omics data analysis, these problems occur at every level being studied. Complicating the problem is that each of the methods for obtaining a particular omics level comes with its own unique technical issues, which can introduce confounding variables. Each omics layer will have its own statistical properties, including data distribution, variance, missing data, and batch effects, which complicate integration [92,93,94,95].

Unpaired data presents a variety of technical and analytical challenges [94,96]. In theory, data across omics layers should be consistently correlated both in presence and abundance. For instance, a gene that is expressed as a transcript should result in the presence and relative abundance of the corresponding protein and its associated metabolites. In practice, this often is not the case. Technical artifacts may affect the identification and quantification of features at different omics layers. By its nature, unpaired omics layers lack shared biological anchors, which must often be inferred by mapping to a shared latent space, such as generating a lower-dimensional space through canonical correlation analysis [94]. This is particularly problematic when mapping a well-developed technology, such as NGS-based transcriptomics, to less well-developed technologies, such as the MS methods used for proteomics. The differences in quality between such datasets can result in missing data (e.g., lost anchors or lack of feature correspondence), which complicates identification of legitimate biological differences between layers introduced by post-transcriptional and post-translational regulation [94]. Much of the existing omics data represents unpaired data, meaning any attempt at reanalysis, meta-analysis, or incorporation of legacy data with new datasets must take the unpaired nature of the data into account. While technical issues with the data are important, biological differences likely represent the primary source of variation between omics layers, particularly at the single-cell level [94]. Significant heterogeneity has been observed in cell cycle [97] and chromatin accessibility [98] between individual cells, and collapsing this data or incorrectly removing it as technical variation can result in a loss of biological information [94].

Another challenge in multi-omics data analysis is different temporal scales across omics layers [1,68,69,75,99]. While the genome is largely static, other omics layers can change over time frames ranging from seconds/minutes (metabolome), minutes/hours (transcriptome), hours/days (proteome), to hours/years (epigenome). While the use of time scale experiments in single omics layer analysis is well-established, integrating multiple omics layers across different time scales is a challenge. Some experiments, such as fluxomics, have the temporal components explicitly incorporated into the model [75], whereas other integrations might require different time course experiments (e.g., more sampling of metabolomics vs. proteome or transcriptome) or the use of more sophisticated analysis tools that explicitly account for temporal variability between layers [100].

While multi-omics studies can be very informative for identifying biomarkers and pathways related to disease [101,102], there exists a well-known gap between generating this data and translating it into practical clinical applications [103,104]. These challenges include biomarker validation, affordability, application across broad and diverse populations, linking of biomarkers to clinically actionable insights, and separating the effects of comorbidities on biomarker abundance [103,104]. From a pharmaceutical sciences perspective, therapies tend to focus on proteins, as they are the more common target for drugs. Given this practical reality and the known disconnect between the transcriptome and the proteome, pharmaceutical sciences researchers tend to gravitate toward proteomic and metabolomic methods. A full multimodal model incorporating multi-omics and health outcomes data may generate a significant number of potential biomarkers, but practical therapies must be at least as accurate, sensitive, practical, and affordable as existing therapies.

The challenges of multi-omics data integration can be summarized as (1) choosing the omics layers most relevant to the analysis to simplify integration, (2) integrating horizontal (i.e., same omics layers across different samples, such as in meta-analysis) and vertical (i.e., true multi-omics analysis) datasets as necessary, (3) understanding the particular statistical properties of each omics layer, including normalization and accounting for missing data, (4) identifying biological anchors between layers for either paired or unpaired data, (5) accounting for temporal differences between omics layers, (6) dimensionality reduction, and (7) identifying layer specific effects (e.g., the unique effects of the proteome) vs. latent effects common to all omics layers.

## 4. Addressing the Challenges in Multi-Omics Analysis

In recent years, many advances have been made to address the challenges of analyzing multi-omics data. These improvements can be generally categorized as (1) instrumentation, (2) methodology/protocols, and (3) algorithms and computation.

Instrumentation Advances—In the early years of the omics era, the scientific and industrial focus was on advances in nucleotide sequencing technologies (e.g., next-gen sequencing, or NGS) [105]. The drive to sequence the human genome and genomes of other model organisms was a major contributor to these advancements [105]. The current focus of NGS technology is the development of highly accurate long-read technologies that will allow, for example, sequencing of entire transcripts and exact quantification of transcript abundance. Recent advancements in LC–MS/GC–MS technology have allowed for corresponding advancements in proteomics and metabolomics methods [105], as well as NGS-based methods for proteome inference. Finally, advancements in single-cell and spatial technologies have advanced considerably in the last ten years, allowing for analysis of millions of individual cells and accurate placement of these cells within their spatial context [37,105]. While emphasis in single-cell methods focused on the transcriptome and epigenome, newer methods allow paired assessment of transcription and surface proteins, with efforts underway to extend this functionality to intracellular proteins.

**Figure 3:**
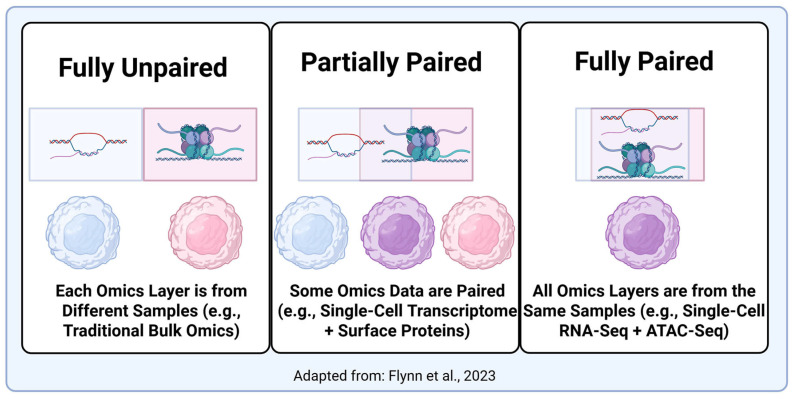
Paired and Unpaired Multi-omics Measurements and Technologies to Analyze the Data Created in BioRender. Hemme, C. (2025) https://BioRender.com/1rcpnd4. Adapted from Flynn et al., 2023 [37].

Methodology and Protocol Advances—Data-Independent Acquisition, or shotgun proteomics, is the proteomics equivalent of shotgun sequencing for nucleic acids and is the standard for proteomics experiments [87]. Similar advancements are improving the ability to identify and quantify metabolomics signatures [34,106]. More accurate whole-ome datasets increase the utility of the datasets and improve the ability to identify cross-level correlations, at the expense of larger and more complex datasets [107]. Improvements in single-cell sample preparation and paired omics data acquisition methods have also contributed to improved datasets (Figure 3, see Strategies for Analyzing Multi-Omics Data below for more details).

Software and Computational Advances—Advances in software and computation have been made to address the challenges in analysis of multi-omics data and networked nature of the integrated data [99]. The transition from bulk to single-cell omics analysis has required modifications to traditional omics data workflows to account for the non-linear nature of single-cell data and the larger and more complex datasets. Identification of cell types from single-cell data requires accurate libraries of gene expression profiles of known cell types. This process has been aided by single-cell atlases created by multiple laboratories that are used as references for subsequent single-cell work. Similarly, accurate LC–MS/GC–MS libraries are required for proper identification of proteins and metabolites [108]. Software such as the Sueret package in R [12] has become the de facto standard for single-cell and spatial data analysis and includes multi-omics data analysis functionality as well. Multiple omics companies have also released software for analysis of multi-omics data (Illumina Connect Multi-omics3, Illumina; Integrated Pathway Analysis6, QIAGEN; Loupe Browser/SpaceRanger7, 10X Genomics) [109]. Many of the tools are designed to address some of the challenges listed in Section 4. All these tools in some fashion consider the networked nature of multi-omics data. Multi-omics data can be modelled as a graph of nodes (biological features) and edges (molecular interactions), making them amenable to methods such as graph neural networks, network inference, similarity-based approaches, and network propagation or diffusion [110]. In tools such as IPA, this data can be further incorporated into curated molecular interaction networks, such as known drug-target interactions [14]. Most commercial packages also have tools for addressing missing data and identifying biological anchors. MOFA+ is designed to identify omics-specific and latent multi-omics properties through matrix deconvolution, and implicitly accounts for some temporal dynamics [13]. Other methods, such as dynamic Bayesian networks [111,112] or ordinary differential equation (ODE) kinetic models, [113,114] can explicitly address temporal differences between layers, allowing for more complex kinetics-based analyses.

Artificial intelligence and machine learning algorithms are increasingly being brought to bear on the challenges of data analysis [92,93,107,115]. ML algorithms have been used to manage the problem of missing data in multi-omics datasets [92,93,95], integrating unpaired data [116], and addressing the challenges of high dimensionality [117] and multimodal datasets [118]. In spatial omics, AI/ML is being used for accurate identification of complex cell shapes such as neurons, which reduces false positives and false negatives associated with improperly assigning omics signals to cells. AI/ML can also be used to correlate omics data with seemingly unrelated cellular properties [119]. For example, researchers have used AI/ML to link Raman spectroscopy to single-cell omics to allow for real-time estimation of gene expression in live cells [120]. AI/ML has also been used to analyze multi-omics data to identify potential drugs for repurposing [121,122,123]. Recent applications employ Graph Neural Networks (GNNs) to model the non-linear interactions between molecular layers, effectively capturing the topology of biological networks. Additionally, Variational Autoencoders (VAEs) are frequently used for dimensionality reduction and data imputation in zero-inflated single-cell datasets. However, the application of these ‘black box’ Deep Learning models introduces challenges regarding interpretability. To address this, attention mechanisms and feature importance ranking are increasingly integrated into models to ensure that AI-derived biomarkers are biologically explainable to clinicians.

The use of AI presents its own challenges, particularly when working with clinical data. Data security and provenance are absolutely essential when working with data that could potentially identify an individual [124,125]. Particularly when working with generative AI, researchers must ensure that patient data is not only anonymized but also not being incorporated into training sets, which could potentially result in the data being made public. Researchers also need to be aware of potential issues in data quality from AI tools [126]. Potential sources of data instability include the following: (1) poorly trained models (e.g., trained on noisy data, insufficient data used for training sets, incorrect assumptions about training data, etc.), (2) inclusion of legacy data that does not meet modern standards of quality control, (3) well-intentioned but inaccurate data (e.g., incorrect or false data from patient surveys), and (4) bias in AI algorithms [126]. At best, this data instability can frustrate data analysis, but at worst could negatively impact patient health and confidentiality.

## 5. Strategies for Analyzing Multi-Omics Data

The nature of multi-omics data presents many analysis challenges, both in acquiring data and in the statistical models used to analyze it. Some common strategies for handling this complexity are listed below.

### 5.1. Paired Data Acquisition

The use of paired data where multiple omics layers are extracted from the same cell or sample greatly simplifies data analysis by reducing the number of batch effects and technical variation that must be managed (Figure 3) [37,127]. For example, 10X Genomics offers kits for simultaneous generation of RNA-seq and ATAC-seq data, and similar methods are being developed by 10X Genomics and other manufacturers for simultaneous generation of transcriptomic and proteomic, or proteomic and metabolomic datasets. Companies such as Illumina also offer protocols for NGS-linked proteomics, which allow for paired proteomics–NGS experiments. Paired datasets are less noisy, have fewer batch effects, and are more easily correlated than traditional unpaired omics data [37].

### 5.2. Integration Architectures (Early, Intermediate, Late)

**Figure 4:**
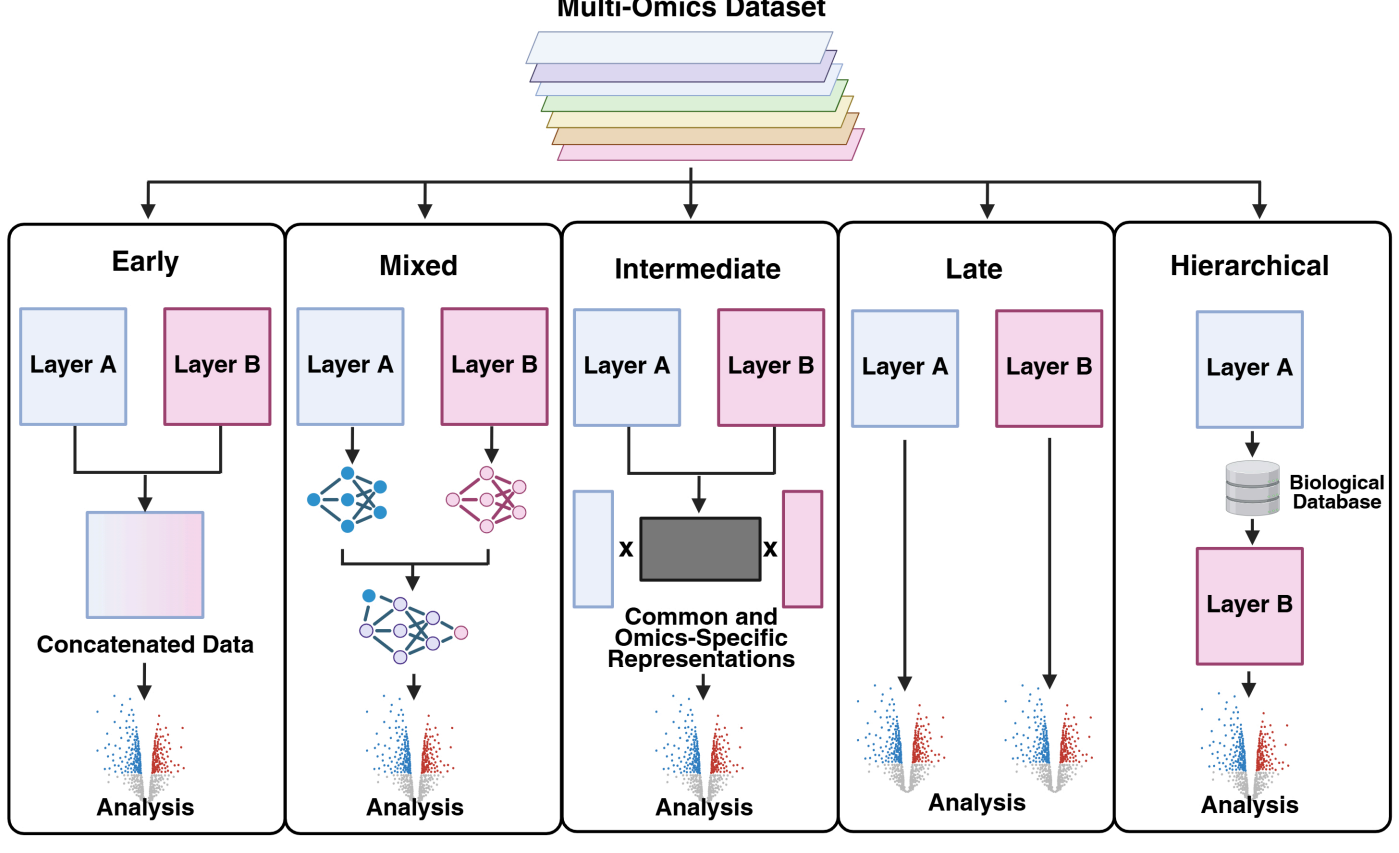
Multi-omics Data Integration Strategies. Created in BioRender. Hemme, C. (2025) https://BioRender.com/08zlp9x. Adapted from Picard et al. 2021 [115].

**Figure 5:**
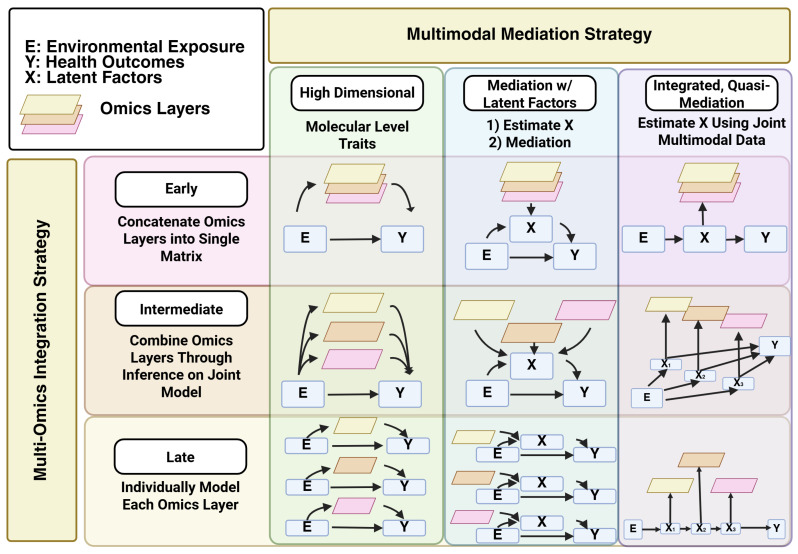
Multimodal Data Integration and Mediation Strategies. Created in BioRender. Hemme, C. (2025) https://BioRender.com/aqjtjyt. Adapted from Goodrich et al., DOI: 10.1016/j.envint.2024.108930 [5].

A central challenge in multi-omics analysis is the effective integration of multiple molecular layers [5,36,115]. The proper strategy greatly depends on the systems being analyzed, their complexity, the quality of the data, and the question being asked. In general, one of three strategies will likely be used (Figure 4 and Figure 5). First, an early-stage integration (concatenation) is most effective when data is paired and/or highly correlated, allowing features from different modalities to be directly linked for each sample. For example, in single-cell multi-omics studies where RNA and ATAC-seq are measured simultaneously from the same nucleus, concatenating these layers into a unified feature space allows for joint dimensionality reduction and clustering, revealing cell states that are coherent across modalities [128]. Tools like Seurat’s [12] weighted nearest neighbor (WNN) and MOFA+ [13] implement this approach to integrate transcriptome and chromatin accessibility data within the same cells [129]. However, the added complexity can make data interpretation difficult and may confuse machine learning (ML) models [130,131]. A *late-stage* strategy where all omics layers are processed individually and then correlated together may be useful for multi-omics experiments with several layers or with messy data. For instance, in a population-scale health study, genomics, metabolomics, and proteomics data may be generated in separate batches. Researchers can first analyze each layer independently (e.g., GWAS for SNPs, differential abundance for metabolites), then perform meta-analysis or pathway enrichment across results to identify convergent biological mechanisms [132]. This approach is implemented in platforms like OmicsNet and PaintOmics, which enable post hoc integration of pre-processed omics results [133]. A *mixed* strategy attempts to reconcile these issues by independently transforming each omics layer into simpler representations prior to integration. This strategy reduces noise and facilitates ML analysis but may still result in lost multi-omics information. An *intermediate* strategy combines omics layers through inference on a joint model and is useful for complex datasets and often assumes a common underlying latent space that reveals the underlying biological mechanisms [115,134,135,136]. In this way, the intermediate strategy can identify a multiomics component common to all omics layers as well as layer-specific components. Finally, a *hierarchical* strategy incorporates prior knowledge of sample biology, such as regulatory networks, to better inform the final integration. All of these methods are described in more detail by Picard et al. [115].

### 5.3. Multimodal Mediation Frameworks

The phenotype of an organism represents a complex interaction between its genotype and environmental factors. While multi-omics alone can provide information about the physical traits associated with a genotype, a more complete picture emerges when additional modalities such as environmental exposures or health outcomes are included [5,137]. Such multimodal experiments require a mediation framework multi-omics to correlate this data with integrated multiomics data [5] (Figure 5). In a hypothetical multimodal experiment, multi-omics data is used to describe how an environmental exposure **E** leads to a health outcome **Y**, with or without a latent factor **X** (Figure 5). A *high-dimensional* strategy treats all potential mediators equally and is useful for biomarker discovery but does not fully account for variation between omics layers. In a *mediation with latent factors* framework, latent factors **X** are estimated, which can allow for measurement of both direct effects of **E** on **Y** and indirect effects mediated by **X**. This strategy is particularly useful for analyzing metabolic pathways. A third strategy, *integrated, quasi-mediation*, involves jointly analyzing environmental exposures and multiomics data and assumes the entire effect of the exposure on the disease is through molecular intermediates [5]. This strategy is useful for identifying subgroups of interest, such as high-risk subject groups. Multi-omics mediation strategies have been used in a variety of contexts, including analyzing the effects of maternal genetic variants on infant health [138], correlating cancer proteogenomics with clinical outcomes [139], and studying the effects of the microbiome on inflammatory bowel disease [140].

## 6. Conclusions

Multi-omics has emerged as a powerful framework for understanding biology at a systems level by integrating genomic, epigenomic, transcriptomic, proteomic, metabolomic, and microbiome data. This holistic approach overcomes the inherent limitations of single-omics studies, enabling deeper insight into the molecular pathways that link genotype to phenotype and accelerating the discovery of biomarkers and therapeutic targets. As multi-omics increasingly incorporates clinical and environmental information, it is poised to play a central role in advancing precision medicine and understanding complex biological processes.

Despite its promise, multi-omics presents significant analytical challenges, including high dimensionality, technical variability, incomplete coverage, and the difficulty of integrating heterogeneous datasets. Recent advancements in high-resolution instrumentation, paired multi-omics protocols, improved sample-processing workflows, and sophisticated computational tools, including AI/ML, are helping to address these obstacles and enhance data quality and interpretability. Continued progress in integration strategies and multimodal analytical frameworks will further strengthen multi-omics as a foundational methodology for systems biology, enabling more rigorous, mechanistic, and clinically meaningful insights.

## Data Availability

No new data were created or analyzed in this perspective article.

## Abbreviations

The following abbreviations are used in this manuscript:

AI	Artificial Intelligence
ATAC-seq	Assay for Transposase-Accessible Chromatin using sequencing
ChIP-seq	Chromatin Immunoprecipitation followed by sequencing
DNA	Deoxyribonucleic Acid
GC	Gas Chromatography
GWAS	Genome-Wide Association Study
LC	Liquid Chromatography
ML	Machine Learning
mRNA	messenger RNA
MS	Mass Spectrometry
NGS	Next-Generation Sequencing
RNA	Ribonucleic Acid
RNA-seq	RNA sequencing
SNPs	Single-Nucleotide Polymorphisms
WNN	Weighted Nearest Neighbor (algorithm in Seurat)

## References

[1] Hasin Y., Seldin M., Lusis A. (2017). Multi-omics approaches to disease. Genome Biol..

[2] Liu J., Yang L., Liu D., Wu Q., Yu Y., Huang X., Li J., Liu S. (2025). The role of multi-omics in biomarker discovery, diagnosis, prognosis, and therapeutic monitoring of tissue repair and regeneration processes. J. Orthop. Translat..

[3] Wang N.N., Cao F., Zhang L.H., Zheng Y.F., Xu D. (2025). Multi-omics: A bridge connecting genotype and phenotype for epilepsy?. Biomark. Res..

[4] Naylor S., Chen J.Y. (2010). Unraveling human complexity and disease with systems biology and personalized medicine. Per. Med..

[5] Goodrich J.A., Wang H., Jia Q., Stratakis N., Zhao Y., Maitre L., Bustamante M., Vafeiadi M., Aung M., Andrušaitytė S. (2024). Integrating Multi-Omics with environmental data for precision health: A novel analytic framework and case study on prenatal mercury induced childhood fatty liver disease. Environ. Int..

[6] Kim J., Woo H.R., Nam H.G. (2016). Toward Systems Understanding of Leaf Senescence: An Integrated Multi-Omics Perspective on Leaf Senescence Research. Mol. Plant.

[B7-biomolecules-16-00271] 7.Illumina. *The Power of Multiomics*; 2022; p. 19. San Diego, CA, USA.

[8] Health, National Human Genome Research Institute National Institutes of Health Multi-Omics for Health and Disease (MOHD). https://www.genome.gov/research-funding/Funded-Programs-Projects/Multi-Omics-for-Health-and-Disease.

[9] Al-Daffaie F.M., Al-Daffaie M.M., Abuhelwa A.Y., Alqudah M.A.Y., Aleidi S.M., El-Huneidi W., Abu-Gharbieh E., Alzoubi K.H., Bustanji Y., Semreen M.H. (2025). Exosomal biomarkers in cancer: Insights from Multi-OMIC approaches. Clin. Chim. Acta.

[10] Chen W., Zhang L., Qi H. (2025). CD44 as a novel therapeutic target in pulmonary arterial hypertension: Insights from multi-omics integration and molecular docking. PLoS ONE.

[11] Gómez F., Pérez-Sánchez N., Macías-Camero A., Núñez R., Contreras N., Cañas J.A., Jiménez-Sánchez I.M., Díez-Echave P., Barbas C., Guéant J.L. (2025). Multi-omics analysis of a prebiotic intervention with pectin in lipid transfer proteins (LTPs) allergic patients. Carbohydr. Polym..

[12] Hao Y., Stuart T., Kowalski M.H., Choudhary S., Hoffman P., Hartman A., Srivastava A., Molla G., Madad S., Fernandez-Granda C. (2024). Dictionary learning for integrative, multimodal and scalable single-cell analysis. Nat. Biotechnol..

[13] Argelaguet R., Arnol D., Bredikhin D., Deloro Y., Velten B., Marioni J.C., Stegle O. (2020). MOFA+: A statistical framework for comprehensive integration of multi-modal single-cell data. Genome Biol..

[14] Krämer A., Green J., Pollard J., Tugendreich S. (2014). Causal analysis approaches in Ingenuity Pathway Analysis. Bioinformatics.

[15] Kohl P., Crampin E.J., Quinn T.A., Noble D. (2010). Systems biology: An approach. Clin. Pharmacol. Ther..

[16] O’Connor J.E., Herrera G., Martínez-Romero A., de Oyanguren F.S., Díaz L., Gomes A., Balaguer S., Callaghan R.C. (2014). Systems Biology and immune aging. Immunol. Lett..

[17] Chuang H.Y., Hofree M., Ideker T. (2010). A decade of systems biology. Annu. Rev. Cell Dev. Biol..

[18] Huang H., Vangay P., McKinlay C.E., Knights D. (2014). Multi-omics analysis of inflammatory bowel disease. Immunol. Lett..

[19] Nie W., Yan L., Lee Y.H., Guha C., Kurland I.J., Lu H. (2016). Advanced mass spectrometry-based multi-omics technologies for exploring the pathogenesis of hepatocellular carcinoma. Mass. Spectrom. Rev..

[20] Williams E.G., Auwerx J. (2015). The Convergence of Systems and Reductionist Approaches in Complex Trait Analysis. Cell.

[21] Chen D., Gyllensten U. (2015). Lessons and implications from association studies and post-GWAS analyses of cervical cancer. Trends Genet..

[22] Buono R.J. (2013). Genome wide association studies (GWAS) and common forms of human epilepsy. Epilepsy Behav..

[23] Sullivan P.F. (2010). The psychiatric GWAS consortium: Big science comes to psychiatry. Neuron.

[24] Prakadan S.M., Shalek A.K., Weitz D.A. (2017). Scaling by shrinking: Empowering single-cell ‘omics’ with microfluidic devices. Nat. Rev. Genet..

[25] Wang D., Bodovitz S. (2010). Single cell analysis: The new frontier in ‘omics’. Trends Biotechnol..

[26] Wang D.C., Wang X. (2017). Systems heterogeneity: An integrative way to understand cancer heterogeneity. Semin. Cell Dev. Biol..

[27] Koutsogiannouli E., Papavassiliou A.G., Papanikolaou N.A. (2013). Complexity in cancer biology: Is systems biology the answer?. Cancer Med..

[28] Yiu T., Chen B., Wang H., Feng G., Fu Q., Hu H. (2025). Transformative advances in single-cell omics: A comprehensive review of foundation models, multimodal integration and computational ecosystems. J. Transl. Med..

[29] Bressan D., Battistoni G., Hannon G.J. (2023). The dawn of spatial omics. Science.

[30] Koppad S., B A., Gkoutos G.V., Acharjee A. (2021). Cloud Computing Enabled Big Multi-Omics Data Analytics. Bioinform. Biol. Insights.

[31] Gao F., Huang K., Xing Y. (2022). Artificial Intelligence in Omics. Genom. Proteom. Bioinform..

[32] Breitling R. (2010). What is systems biology?. Front. Physiol..

[33] Sidoli S., Kulej K., Garcia B.A. (2017). Why proteomics is not the new genomics and the future of mass spectrometry in cell biology. J. Cell Biol..

[34] Johnson C.H., Gonzalez F.J. (2012). Challenges and opportunities of metabolomics. J. Cell Physiol..

[35] Workman J. (2024). Advancements and Emerging Techniques in Mass Spectrometry: A Comprehensive Review. LCGC Supplements.

[36] Subramanian I., Verma S., Kumar S., Jere A., Anamika K. (2020). Multi-omics Data Integration, Interpretation, and Its Application. Bioinform. Biol. Insights.

[37] Flynn E., Almonte-Loya A., Fragiadakis G.K. (2023). Single-Cell Multiomics. Annu. Rev. Biomed. Data Sci..

[38] Babu M., Snyder M. (2023). Multi-Omics Profiling for Health. Mol. Cell. Proteom..

[39] Morabito A., De Simone G., Pastorelli R., Brunelli L., Ferrario M. (2025). Algorithms and tools for data-driven omics integration to achieve multilayer biological insights: A narrative review. J. Transl. Med..

[40] Collins F.S., Morgan M., Patrinos A. (2003). The Human Genome Project: Lessons from large-scale biology. Science.

[41] Frazer K.A., Ballinger D.G., Cox D.R., Hinds D.A., Stuve L.L., Gibbs R.A., Belmont J.W., Boudreau A., Hardenbol P., Leal S.M. (2007). A second generation human haplotype map of over 3.1 million SNPs. Nature.

[42] Proctor L.M., Creasy H.H., Fettweis J.M., Lloyd-Price J., Mahurkar A., Zhou W., Buck G.A., Snyder M.P., Strauss J.F., Weinstock G.M. (2019). The Integrative Human Microbiome Project. Nature.

[43] Waterston R.H., Lindblad-Toh K., Birney E., Rogers J., Abril J.F., Agarwal P., Agarwala R., Ainscough R., Alexandersson M., An P. (2002). Initial sequencing and comparative analysis of the mouse genome. Nature.

[44] Celniker S.E., Rubin G.M. (2003). The Drosophila melanogaster genome. Annu. Rev. Genom. Hum. Genet..

[45] Jeffries A.M., Yu T., Ziegenfuss J.S., Tolles A.K., Baer C.E., Sotelo C.B., Kim Y., Weng Z., Lodato M.A. (2025). Single-cell transcriptomic and genomic changes in the ageing human brain. Nature.

[46] Xue J., Schmidt S.V., Sander J., Draffehn A., Krebs W., Quester I., De Nardo D., Gohel T.D., Emde M., Schmidleithner L. (2014). Transcriptome-based network analysis reveals a spectrum model of human macrophage activation. Immunity.

[47] Ma S., Zhang Y. (2020). Profiling chromatin regulatory landscape: Insights into the development of ChIP-seq and ATAC-seq. Mol. Biomed..

[48] Farsetti A., Illi B., Gaetano C. (2023). How epigenetics impacts on human diseases. Eur. J. Intern. Med..

[49] Lister R., Pelizzola M., Dowen R.H., Hawkins R.D., Hon G., Tonti-Filippini J., Nery J.R., Lee L., Ye Z., Ngo Q.M. (2009). Human DNA methylomes at base resolution show widespread epigenomic differences. Nature.

[50] Hewick R.M., Lu Z., Wang J.H., Smith R.D., Veenstra T.D. (2003). Proteomics in Drug Discovery. Advances in Protein Chemistry.

[51] Wang S., Osgood A.O., Chatterjee A. (2022). Uncovering post-translational modification-associated protein-protein interactions. Curr. Opin. Struct. Biol..

[52] Palukuri M.V., Patil R.S., Marcotte E.M. (2023). Molecular complex detection in protein interaction networks through reinforcement learning. BMC Bioinform..

[53] Dayon L., Cominetti O., Affolter M. (2022). Proteomics of human biological fluids for biomarker discoveries: Technical advances and recent applications. Expert. Rev. Proteom..

[54] Rozanova S., Barkovits K., Nikolov M., Schmidt C., Urlaub H., Marcus K. (2021). Quantitative Mass Spectrometry-Based Proteomics: An Overview. Methods Mol. Biol..

[55] Sun Y., Zhang X., Hang D., Lau H.C., Du J., Liu C., Xie M., Pan Y., Wang L., Liang C. (2024). Integrative plasma and fecal metabolomics identify functional metabolites in adenoma-colorectal cancer progression and as early diagnostic biomarkers. Cancer Cell.

[56] Jové M., Mota-Martorell N., Torres P., Portero-Otin M., Ferrer I., Pamplona R. (2021). New insights into human prefrontal cortex aging with a lipidomics approach. Expert. Rev. Proteom..

[57] Bua R.O., Messina A., Sturiale L., Barone R., Garozzo D., Palmigiano A. (2021). N-Glycomics of Human Erythrocytes. Int. J. Mol. Sci..

[58] Moiz B., Li A., Padmanabhan S., Sriram G., Clyne A.M. (2022). Isotope-Assisted Metabolic Flux Analysis: A Powerful Technique to Gain New Insights into the Human Metabolome in Health and Disease. Metabolites.

[59] Nyholm L., Koziol A., Marcos S., Botnen A.B., Aizpurua O., Gopalakrishnan S., Limborg M.T., Gilbert M.T.P., Alberdi A. (2020). Holo-Omics: Integrated Host-Microbiota Multi-omics for Basic and Applied Biological Research. iScience.

[60] Kunath B.J., De Rudder C., Laczny C.C., Letellier E., Wilmes P. (2024). The oral-gut microbiome axis in health and disease. Nat. Rev. Microbiol..

[61] Carmona-Cruz S., Orozco-Covarrubias L., Sáez-de-Ocariz M. (2022). The Human Skin Microbiome in Selected Cutaneous Diseases. Front. Cell. Infect. Microbiol..

[62] Lhoumaud P., Sethia G., Izzo F., Sakellaropoulos T., Snetkova V., Vidal S., Badri S., Cornwell M., Di Giammartino D.C., Kim K.T. (2019). EpiMethylTag: Simultaneous detection of ATAC-seq or ChIP-seq signals with DNA methylation. Genome Biol..

[63] Spektor R., Tippens N.D., Mimoso C.A., Soloway P.D. (2019). methyl-ATAC-seq measures DNA methylation at accessible chromatin. Genome Res..

[64] Sun N., Victor M.B., Park Y.P., Xiong X., Scannail A.N., Leary N., Prosper S., Viswanathan S., Luna X., Boix C.A. (2023). Human microglial state dynamics in Alzheimer’s disease progression. Cell.

[65] Zhang D., Deng Y., Kukanja P., Agirre E., Bartosovic M., Dong M., Ma C., Ma S., Su G., Bao S. (2023). Spatial epigenome-transcriptome co-profiling of mammalian tissues. Nature.

[66] Samih A., de Moura Ferreira M.A., Nikoloski Z. (2026). Gene expression and protein abundance: Just how associated are these molecular traits?. Biotechnol. Adv..

[67] Spielmann M., Mundlos S. (2016). Looking beyond the genes: The role of non-coding variants in human disease. Human. Mol. Genet..

[68] Liu Y., Beyer A., Aebersold R. (2016). On the Dependency of Cellular Protein Levels on mRNA Abundance. Cell.

[69] Schwanhäusser B., Busse D., Li N., Dittmar G., Schuchhardt J., Wolf J., Chen W., Selbach M. (2011). Global quantification of mammalian gene expression control. Nature.

[70] Fagerberg L., Hallström B.M., Oksvold P., Kampf C., Djureinovic D., Odeberg J., Habuka M., Tahmasebpoor S., Danielsson A., Edlund K. (2014). Analysis of the human tissue-specific expression by genome-wide integration of transcriptomics and antibody-based proteomics. Mol. Cell. Proteom..

[71] Chen Z.Z., Gerszten R.E. (2020). Metabolomics and Proteomics in Type 2 Diabetes. Circ. Res..

[72] van Vugt M., Finan C., Chopade S., Providencia R., Bezzina C.R., Asselbergs F.W., van Setten J., Schmidt A.F. (2024). Integrating metabolomics and proteomics to identify novel drug targets for heart failure and atrial fibrillation. Genome Med..

[73] Alghamdi N., Chang W., Dang P., Lu X., Wan C., Gampala S., Huang Z., Wang J., Ma Q., Zang Y. (2021). A graph neural network model to estimate cell-wise metabolic flux using single-cell RNA-seq data. Genome Res..

[74] Brejchova K., Rahm M., Benova A., Domanska V., Reyes-Gutierez P., Dzubanova M., Trubacova R., Vondrackova M., Cajka T., Tencerova M. (2025). Uncovering mechanisms of thiazolidinediones on osteogenesis and adipogenesis using spatial fluxomics. Metabolism.

[75] Hackett S.R., Zanotelli V.R., Xu W., Goya J., Park J.O., Perlman D.H., Gibney P.A., Botstein D., Storey J.D., Rabinowitz J.D. (2016). Systems-level analysis of mechanisms regulating yeast metabolic flux. Science.

[76] Ferkingstad E., Sulem P., Atlason B.A., Sveinbjornsson G., Magnusson M.I., Styrmisdottir E.L., Gunnarsdottir K., Helgason A., Oddsson A., Halldorsson B.V. (2021). Large-scale integration of the plasma proteome with genetics and disease. Nat. Genet..

[77] Mishra A., Malik R., Hachiya T., Jürgenson T., Namba S., Posner D.C., Kamanu F.K., Koido M., Le Grand Q., Shi M. (2022). Stroke genetics informs drug discovery and risk prediction across ancestries. Nature.

[78] Daskalakis N.P., Iatrou A., Chatzinakos C., Jajoo A., Snijders C., Wylie D., DiPietro C.P., Tsatsani I., Chen C.Y., Pernia C.D. (2024). Systems biology dissection of PTSD and MDD across brain regions, cell types, and blood. Science.

[79] Lloyd-Price J., Arze C., Ananthakrishnan A.N., Schirmer M., Avila-Pacheco J., Poon T.W., Andrews E., Ajami N.J., Bonham K.S., Brislawn C.J. (2019). Multi-omics of the gut microbial ecosystem in inflammatory bowel diseases. Nature.

[80] Lavelle A., Sokol H. (2020). Gut microbiota-derived metabolites as key actors in inflammatory bowel disease. Nat. Rev. Gastroenterol. Hepatol..

[81] Verma A., Inslicht S.S., Bhargava A. (2024). Gut-Brain Axis: Role of Microbiome, Metabolomics, Hormones, and Stress in Mental Health Disorders. Cells.

[82] Allegaert K. (2022). Integrated in a systems pharmacology approach, pharmacogenetics holds the promise for personalized medicine in neonates. Pharmacogenomics.

[83] Zhao Q., Chen Y., Huang W., Zhou H., Zhang W. (2023). Drug-microbiota interactions: An emerging priority for precision medicine. Signal Transduct. Target. Ther..

[84] Antman E., Weiss S., Loscalzo J. (2012). Systems pharmacology, pharmacogenetics, and clinical trial design in network medicine. Wiley Interdiscip. Rev. Syst. Biol. Med..

[85] Bloomingdale P., Karelina T., Cirit M., Muldoon S.F., Baker J., McCarty W.J., Geerts H., Macha S. (2021). Quantitative systems pharmacology in neuroscience: Novel methodologies and technologies. CPT Pharmacomet. Syst. Pharmacol..

[86] Luo Y., Zhao C., Chen F. (2024). Multiomics Research: Principles and Challenges in Integrated Analysis. Biodes Res..

[87] Movassaghi C.S., Sun J., Jiang Y., Turner N., Chang V., Chung N., Chen R.J., Browne E.N., Lin C., Schweppe D.K. (2025). Recent Advances in Mass Spectrometry-Based Bottom-Up Proteomics. Anal. Chem..

[88] Ugidos M., Nueda M.J., Prats-Montalbán J.M., Ferrer A., Conesa A., Tarazona S. (2022). MultiBaC: An R package to remove batch effects in multi-omic experiments. Bioinformatics.

[89] Molania R., Foroutan M., Gagnon-Bartsch J.A., Gandolfo L.C., Jain A., Sinha A., Olshansky G., Dobrovic A., Papenfuss A.T., Speed T.P. (2023). Removing unwanted variation from large-scale RNA sequencing data with PRPS. Nat. Biotechnol..

[90] Scherer A. (2009). Batch Effects and Noise in Microarray Experiments: Sources and Solutions.

[91] Misra B.B. (2020). Data normalization strategies in metabolomics: Current challenges, approaches, and tools. Eur. J. Mass Spectrom..

[92] Baião A.R., Cai Z., Poulos R.C., Robinson P.J., Reddel R.R., Zhong Q., Vinga S., Gonçalves E. (2025). A technical review of multi-omics data integration methods: From classical statistical to deep generative approaches. Brief. Bioinform..

[93] Flores J.E., Claborne D.M., Weller Z.D., Webb-Robertson B.M., Waters K.M., Bramer L.M. (2023). Missing data in multi-omics integration: Recent advances through artificial intelligence. Front. Artif. Intell..

[94] Rautenstrauch P., Vlot A.H.C., Saran S., Ohler U. (2022). Intricacies of single-cell multi-omics data integration. Trends Genet..

[95] Das S., Mukhopadhyay I. (2021). TiMEG: An integrative statistical method for partially missing multi-omics data. Sci. Rep..

[96] Abir A.R., Dip S.A., Zhang L. (2025). UnCOT-AD: Unpaired Cross-Omics Translation Enables Multi-Omics Integration for Alzheimer’s Disease Prediction. Brief. Bioinform..

[97] Movasat H., Giacopino E., Shahdoost A., Dorri Nokoorani Y., Abrbekouh A.H., Tahamtani Y., Shakiba N. (2025). A systems view of cellular heterogeneity: Unlocking the “wheel of fate”. Cell Syst..

[98] Muto Y., Wilson P.C., Ledru N., Wu H., Dimke H., Waikar S.S., Humphreys B.D. (2021). Single cell transcriptional and chromatin accessibility profiling redefine cellular heterogeneity in the adult human kidney. Nat. Commun..

[99] Sanches P.H.G., de Melo N.C., Porcari A.M., de Carvalho L.M. (2024). Integrating Molecular Perspectives: Strategies for Comprehensive Multi-Omics Integrative Data Analysis and Machine Learning Applications in Transcriptomics, Proteomics, and Metabolomics. Biology.

[100] Ritchie M.D., Holzinger E.R., Li R., Pendergrass S.A., Kim D. (2015). Methods of integrating data to uncover genotype-phenotype interactions. Nat. Rev. Genet..

[101] FDA-NIH Biomarker Working Group (2016). BEST (Biomarkers, EndpointS, and Other Tools) Resource [Internet].

[102] WHO International Programme on Chemical Safety Biomarkers in Risk Assessment: Validity and Validation. https://inchem.org/documents/ehc/ehc/ehc222.htm.

[103] Herzog C.M.S., Goeminne L.J.E., Poganik J.R., Barzilai N., Belsky D.W., Betts-LaCroix J., Chen B.H., Chen M., Cohen A.A., Cummings S.R. (2024). Challenges and recommendations for the translation of biomarkers of aging. Nat. Aging.

[104] Schöll M., Verberk I.M.W., del Campo M., Delaby C., Therriault J., Chong J.R., Palmqvist S., Alcolea D. (2024). Challenges in the practical implementation of blood biomarkers for Alzheimer’s disease. Lancet Healthy Longev..

[105] Dai X., Shen L. (2022). Advances and Trends in Omics Technology Development. Front. Med..

[106] Titkare N., Chaturvedi S., Borah S., Sharma N. (2024). Advances in mass spectrometry for metabolomics: Strategies, challenges, and innovations in disease biomarker discovery. Biomed. Chromatogr..

[107] Vidanagamachchi S.M., Waidyarathna K. (2024). Opportunities, challenges and future perspectives of using bioinformatics and artificial intelligence techniques on tropical disease identification using omics data. Front. Digit. Health.

[108] Bittremieux W., Wang M., Dorrestein P.C. (2022). The critical role that spectral libraries play in capturing the metabolomics community knowledge. Metabolomics.

[109] Hayes C.N., Nakahara H., Ono A., Tsuge M., Oka S. (2024). From Omics to Multi-Omics: A Review of Advantages and Tradeoffs. Genes.

[110] Jiang W., Ye W., Tan X., Bao Y.J. (2025). Network-based multi-omics integrative analysis methods in drug discovery: A systematic review. BioData Min..

[111] Sachs K., Perez O., Pe’er D., Lauffenburger D.A., Nolan G.P. (2005). Causal Protein-Signaling Networks Derived from Multiparameter Single-Cell Data. Science.

[112] Sato T. (2026). Computational Framework for Causal Inference in Molecular Dynamics Analysis of Lipid–Protein Interactions. J. Chem. Inf. Model..

[113] Borsari B., Frank M., Wattenberg E.S., Xu K., Liu S.X., Yu X., Gerstein M. (2025). The chronODE framework for modelling multi-omic time series with ordinary differential equations and machine learning. Nat. Commun..

[114] Toumpe I., Choudhury S., Hatzimanikatis V., Miskovic L. (2025). The Dawn of High-Throughput and Genome-Scale Kinetic Modeling: Recent Advances and Future Directions. ACS Synth. Biol..

[115] Picard M., Scott-Boyer M.-P., Bodein A., Périn O., Droit A. (2021). Integration strategies of multi-omics data for machine learning analysis. Comput. Struct. Biotechnol. J..

[116] Kan Y., Qi Y., Zhang Z., Liang X., Wang W., Jin S. (2025). Integration of unpaired single cell omics data by deep transfer graph convolutional network. PLoS Comput. Biol..

[117] Zhou Y., Geng P., Zhang S., Xiao F., Cai G., Chen L., Lu Q. (2024). Multimodal functional deep learning for multiomics data. Brief. Bioinform..

[118] Lin X., Jiang S., Gao L., Wei Z., Wang J. (2024). MultiSC: A deep learning pipeline for analyzing multiomics single-cell data. Brief. Bioinform..

[119] Coleman K., Schroeder A., Loth M., Zhang D., Park J.H., Sung J.Y., Blank N., Cowan A.J., Qian X., Chen J. (2025). Resolving tissue complexity by multimodal spatial omics modeling with MISO. Nat. Methods.

[120] Kobayashi-Kirschvink K.J., Comiter C.S., Gaddam S., Joren T., Grody E.I., Ounadjela J.R., Zhang K., Ge B., Kang J.W., Xavier R.J. (2024). Prediction of single-cell RNA expression profiles in live cells by Raman microscopy with Raman2RNA. Nat. Biotechnol..

[121] Jurj E.D., Colibășanu D., Vasii S.O., Suciu L., Dehelean C.A., Udrescu L. (2025). Redefining Breast Cancer Care by Harnessing Computational Drug Repositioning. Medicina.

[122] Huang J., Gao J., Chen Q. (2025). An Interpretable Deep Learning and Molecular Docking Framework for Repurposing Existing Drugs as Inhibitors of SARS-CoV-2 Main Protease. Molecules.

[123] Wei S., Sasi C., Piepenbrock J., Huynen M.A., t Hoen P.A.C. (2025). The use of knowledge graphs for drug repurposing: From classical machine learning algorithms to graph neural networks. Comput. Biol. Med..

[124] Ahmed M., Dar A.R., Helfert M., Khan A., Kim J. (2023). Data Provenance in Healthcare: Approaches, Challenges, and Future Directions. Sensors.

[125] Simjanoska Misheva M., Shahpaski D., Dobreva J., Bukovec D., Gjorgjioski B., Nikolov M., Frtunikj D., Lameski P., Aliu A., Mishev K. (2025). AI Act Compliance Within the MyHealth@EU Framework: Tutorial. J. Med. Internet Res..

[126] Carini C., Seyhan A.A. (2024). Tribulations and future opportunities for artificial intelligence in precision medicine. J. Transl. Med..

[127] Huizing G.-J., Deutschmann I.M., Peyré G., Cantini L. (2023). Paired single-cell multi-omics data integration with Mowgli. Nat. Commun..

[128] Xu F., Cong P., Lu Z., Shi L., Xiong L., Zhao G. (2023). Integration of ATAC-Seq and RNA-Seq identifies key genes and pathways involved in the neuroprotection of S-adenosylmethionine against perioperative neurocognitive disorder. Comput. Struct. Biotechnol. J..

[129] Hao Y., Hao S., Andersen-Nissen E., Mauck W.M., Zheng S., Butler A., Lee M.J., Wilk A.J., Darby C., Zager M. (2021). Integrated analysis of multimodal single-cell data. Cell.

[130] Lee B., Zhang S., Poleksic A., Xie L. (2020). Heterogeneous multi-layered network model for omics data integration and analysis. Front. Genet..

[131] Ma T., Zhang A. (2016). Omics informatics: From scattered individual software tools to integrated workflow management systems. IEEE/ACM Trans. Comput. Biol. Bioinform..

[132] Ogris C., Hu Y., Arloth J., Müller N.S. (2021). Versatile knowledge guided network inference method for prioritizing key regulatory factors in multi-omics data. Sci. Rep..

[133] Papadaki E., Kakkos I., Vlamos P., Petropoulou O., Miloulis S.T., Palamas S., Vrahatis A.G. (2025). Recent Web Platforms for Multi-Omics Integration Unlocking Biological Complexity. Appl. Sci..

[134] Cao Z.-J., Gao G. (2022). Multi-omics single-cell data integration and regulatory inference with graph-linked embedding. Nat. Biotechnol..

[135] Liu T., Salguero P., Petek M., Martinez-Mira C., Balzano-Nogueira L., Ramšak Ž., McIntyre L., Gruden K., Tarazona S., Conesa A. (2022). PaintOmics 4: New tools for the integrative analysis of multi-omics datasets supported by multiple pathway databases. Nucleic Acids Res..

[136] Zhou G., Pang Z., Lu Y., Ewald J., Xia J. (2022). OmicsNet 2.0: A web-based platform for multi-omics integration and network visual analytics. Nucleic Acids Res..

[137] Maitre L., Bustamante M., Hernández-Ferrer C., Thiel D., Lau C.-H.E., Siskos A.P., Vives-Usano M., Ruiz-Arenas C., Pelegrí-Sisó D., Robinson O. (2022). Multi-omics signatures of the human early life exposome. Nat. Commun..

[138] Gill N.P., Kuang A., Scholtens D.M. (2025). Multi-omics mediation pipeline reveals differential pathways of maternal SNPs affecting newborn adiposity outcomes. BMC Genom. Data.

[139] Huang L., Long J.P., Irajizad E., Doecke J.D., Do K.-A., Ha M.J. (2023). A unified mediation analysis framework for integrative cancer proteogenomics with clinical outcomes. Bioinformatics.

[140] Jiang H., Miao X., Thairu M.W., Beebe M., Grupe D.W., Davidson R.J., Handelsman J., Sankaran K. (2025). Multimedia: Multimodal mediation analysis of microbiome data. Microbiol. Spectr..

